# Control of *Scaphoideus titanus* with Natural Products in Organic Vineyards

**DOI:** 10.3390/insects8040129

**Published:** 2017-12-16

**Authors:** Federico Tacoli, Nicola Mori, Alberto Pozzebon, Elena Cargnus, Sarah Da Vià, Pietro Zandigiacomo, Carlo Duso, Francesco Pavan

**Affiliations:** 1 Department of Agricultural, Food, Environmental and Animal Sciences, University of Udine, via delle Scienze 206, Udine 33100, Italy; elena.cargnus@uniud.it (E.C.); pietro.zandigiacomo@uniud.it (P.Z.); francesco.pavan@uniud.it (F.P.); 2 Department of Agronomy, Food, Natural Resources, Animals and Environment (DAFNAE), University of Padova, via dell’Università 16, Agripolis, Legnaro 35020, Padova, Italy; nicola.mori@unipd.it (N.M.); alberto.pozzebon@unipd.it (A.P.); sarah.davia@hotmail.com (S.D.V.); carlo.duso@unipd.it (C.D.)

**Keywords:** flavescence dorée, *Scaphoideus titanus*, vector control, natural substances, kaolin, pyrethrins, organic viticulture

## Abstract

The leafhopper *Scaphoideus titanus* is the vector of ‘*Candidatus* Phytoplasma vitis’, the causal agent of Flavescence dorée (FD) a key disease for European viticulture. In organic vineyards, the control of *S. titanus* relies mostly on the use of pyrethrins that have suboptimal efficacy. During 2016, three field trials were conducted to evaluate the efficacy of kaolin, orange oil, insecticidal soap and spinosad against *S. titanus* nymphs, in comparison with pyrethrins. The activity of kaolin was evaluated also in the laboratory. In all field trials, kaolin had an efficacy against nymphs comparable to pyrethrins, while the other products were not effective. Laboratory results confirmed that kaolin increased nymph mortality. In organic vineyards, kaolin and pyrethrins are valuable tools in the management of FD. Nevertheless, their efficacy is lower compared to that of the synthetic insecticides used in conventional viticulture. Therefore, further research should be conducted in order to identify alternatives to synthetic insecticides for *S. titanus* control in the context of organic viticulture.

## 1. Introduction

The leafhopper *Scaphoideus titanus* Ball (Hemiptera: Cicadellidae) is the vector of ‘*Candidatus* Phytoplasma vitis’, the causal agent of Flavescence dorée (FD), which is a Grapevine Yellows Disease (GYD) that causes severe damage in European vineyards [1,2,3,4].

Flavescence-dorée phytoplasma is a quarantine disease in the European and Mediterranean Plant Protection Organization (EPPO) region [5] and control measures are mandatory in some European countries [6]. The FD control strategy in vineyards relies mostly on *S. titanus* control and on the roguing of symptomatic grapevines, from which the vector can acquire the phytoplasma [6,7,8,9]. However, since insecticide applications are poorly effective against infected adults migrating into vineyards [10,11,12], the strategy adopted is effective only if external sources of infected individuals (i.e., infected vineyards, both abandoned or cultivated but untreated against *S. titanus*, and wild American grapevines growing in hedgerows and groves) are previously removed.

In Italian conventional vineyards, one to two insecticide applications against *S. titanus* can keep the populations of this vector and the percentage of FD-symptomatic grapevines at acceptable levels [11,13,14]. Infected grapevines can also die or recover, but this latter capability varies across cultivars. In cultivars with a high incidence of recovery, the chemical control of *S. titanus* can bring the percentage of symptomatic grapevines back to acceptable levels without their roguing [15]. The high efficacy of insecticide applications in vineyards is due to the biology of *S. titanus*: (i) it is monophagous on *Vitis* sp. plants, therefore external sources can be easily removed, and (ii) it completes only one generation a year after being overwintered as eggs [6,16,17]. The control strategy aims to kill nymphs at the completion of the latency period, that is, before they have reached the fourth-fifth instars, which is the point that they become infective [13,18]. The need for more than one insecticide application derives from the prolonged egg-hatching period, which lasts for over 45 days [17]. The timing of insecticide applications is usually based on nymph samplings. In Italy, the first insecticide application is performed at the appearance of third instar nymphs and the second application occurs after two or three weeks to kill the nymphs hatched after the first application [13]. 

In conventional vineyards, an effective control of *S. titanus* nymphs is ensured by the use of organophosphates, pyrethroids and neonicotinoids [19,20,21,22,23]. All these insecticides are characterized by a long residual activity. 

In organic vineyards, the control of *S. titanus* is difficult and often higher population levels are observed as compared to conventional vineyards, despite several insecticide applications [11]. The most effective insecticides in organic viticulture are pyrethrins, which are used alone or in combination with piperonyil butoxide, mineral oil or sesame oil [21,22,24,25,26,27,28,29]. However, the effectiveness of pyrethrins is lower than synthetic insecticides [11,22], because the former exert a marked knock-down effect when nymphs are directly hit, but the effectiveness declines to low levels when nymphs are kept in contact with fresh residues [26]. The absence of residual activity in pyrethrins mandates many applications to cover the entire egg-hatching period adequately. Mineral oil, spinosad, azadirachtin and *Beauveria bassiana* are considered less effective than pyrethrins or totally ineffective [22,24,25,26,27,28]. The need for repeated pyrethrin applications to overcome the low persistence of this class of insecticides may result in detrimental effects on predatory mites of the Phytoseiidae family [26,28,30]. Considering both the low efficacy and the toxicity to non-target organisms of pyrethrins, the identification of other natural products is necessary.

Recently, natural products such as kaolin, essential oils and insecticidal soap (i.e., potassium salts of fatty acids) have been found to be effective against leafhoppers or other Hemiptera [31,32,33,34,35]. In particular, a high efficacy of kaolin against the leafhoppers *Empoasca vitis* (Göthe) and *Zygina rhamni* Ferrari (Hemiptera: Cicadellidae) was observed both in vineyards and in the laboratory [32]. In the laboratory, plant essential oils have been found to exhibit a toxic activity against mealybugs (Hemiptera: Pseudococcidae) [33]. Insecticidal soap was effective in the control of some Pentatomidae under field conditions [34] and *Halyomorpha halys* (Stål) (Hemiptera: Pentatomidae) in the laboratory [35]. In the present study, the efficacy of kaolin, orange oil, insecticidal soap and spinosad against *S. titanus* nymphs was compared to pyrethrins in field trials. To evaluate if kaolin had any effects on egg laying by females, nymph population levels in the kaolin and control were assessed in the year following the kaolin applications. The activity of kaolin on nymphs was also evaluated in the laboratory.

## 2. Materials and Methods

### 2.1. Field Trials 

In 2016, three field trials were carried out in vineyards located in north-eastern Italy to evaluate the efficacy of five natural products (Table 1) against *S. titanus* in comparison to an untreated control. Vineyard I (Togliano, Udine district, 46°06′45″ N, 13°24′40″ E, 140 m a.s.l., cultivar Merlot) is a 15-yr-old conventional vineyard with grapevines growing under the Guyot training system with distances between and along the rows of 2.4 m and 0.7 m, respectively. Vineyard II (Nimis, Udine district, 46°11′34″ N, 13°15′42″ E, 200 m a.s.l., cultivar Verduzzo Friulano) is a 15-yr-old conventional vineyard with grapevines growing under the Guyot training system with distances between and along the rows of 2.9 m and 0.8 m, respectively. Vineyard III (Lonigo, Vicenza district, 45°24′04″ N, 11°23′26″ E, 31 m a.s.l., cultivar Garganega) is a 20-yr-old organic vineyard with grapevines growing under the “Pergola” training system with distances between and along the rows of 4.0 m and 1.0 m, respectively. In all vineyards, a standard fungicide program was followed and no insecticides were applied during the growing season.

In all trials, a randomized block design with four replicates was adopted. Each block (row) was divided into 6 plots of 20 (vineyard I) or 16 (vineyard II) or 15 (vineyard III) grapevines and product applications were planned as described in Table 1. The timing of the applications was based on the appearance of different *S. titanus* instar nymphs, in particular: (A) first-instar nymphs (26 May in vineyard I, 25 May in vineyard II and 26 May in vineyard III); (B) second-instar nymphs (1 June in vineyard I, 3 June in vineyard II and 1 June in vineyard III); (C) third-instar nymphs (8 June in vineyard I, 8 June in vineyard II and 10 June in vineyard III); and (D) fourth-instar nymphs (14 June in vineyard I, 14 June in vineyard II and 15 June in vineyard III). All products were applied using a backpack sprayer (M1200, Cifarelli s.p.a., Voghera, PV, Italy) at a rate of 1000 L/ha spraying the canopy and the suckers growing along the vertical trunk. 

In all vineyards, *S. titanus* nymphs were sampled before application timing A (25 May in vineyard I, 3 June in vineyard II and 26 of May in vineyard III) and weekly up to one week after application timing D (i.e., 28 June in vineyard I, 6 July in vineyard II and 30 June in vineyard III). Sampling was conducted on the 10 central grapevines of each plot. *Scaphoideus titanus* nymphs were counted on sucker leaves. Suckers were chosen as sampling units because in spring they host the highest nymph density [13,36]. In vineyards I and II, five sucker leaves per grapevine were sampled for a total of 200 leaves per treatment. In vineyard III, all leaves of 10 suckers per plot were sampled for a total of 40 suckers per treatment being the population density too low to use leaf as sampling unit.

In vineyards I and II, *S. titanus* nymphs were also sampled in early June of 2017 in the plots that in 2016 belonged to the control and the kaolin. In vineyard III, this sampling was not done because a late frost heavily damaged suckers.

### 2.2. Laboratory Bioassay

A laboratory bioassay was carried out to evaluate the efficacy of kaolin against *S. titanus* nymphs. Mortality of first-instar and second-instar nymphs was compared in two treatments: (i) nymphs placed on kaolin-treated leaves (kaolin); and (ii) nymphs placed on water-treated leaves (control). The sample size was equal to 50 leaves per treatment. For this purpose, 100 insecticide-free grapevine leaves were collected from vineyard I. In the laboratory, each leaf was visually checked to ensure the absence of *S. titanus* individuals. Fifty leaves were sprayed with kaolin and 50 with water, and each were inserted individually into transparent self-sealing plastic bags (20 × 33 cm). The kaolin application was done at a 4% *W*:*V* (Surround WP:water) concentration with a hand sprayer to run-off. The *S. titanus* nymphs used in the bioassay were collected from insecticide-free leaves picked in the same vineyard. First- and second-instar nymphs were randomly chosen on these leaves and a single individual was induced to move onto each bagged leaf with a brush. After 1, 2, 3, 6 and 9 days from the beginning of the bioassay, the bags were checked to note whether nymphs were alive or dead. 

### 2.3. Statistical Analyses

Statistical analyses were performed with Microsoft Excel 2013 for Windows (Microsoft Corporation 2013, Redmond, WA, USA) and SAS (v 9.4, SAS Institute, Cary, NC, USA). 

Data collected in the field trials in 2016 were analyzed using mixed linear models performed with the PROC MIXED (SAS Institute 9.4). In modelling treatment, time and their interactions were considered as sources of variation and F tests were used to evaluate their effects (α = 0.05). Numbers of *S. titanus* nymphs were considered as response variable with repeated measures made at different times, i.e. sampling dates. Treatments were compared using a *t*-test to the least-square means with Bonferroni adjustment of the *p*-values (α = 0.05). Data collected in the spring of 2017 were compared with a *t*-test. Data were log (*x* + 1) transformed prior to the analyses.

Data collected in the laboratory bioassay were compared with a Fisher’s exact test and the mortality percentage of nymphs was calculated following Abbott [37]. 

## 3. Results

### 3.1. Field Trials 

**Figure 1:**
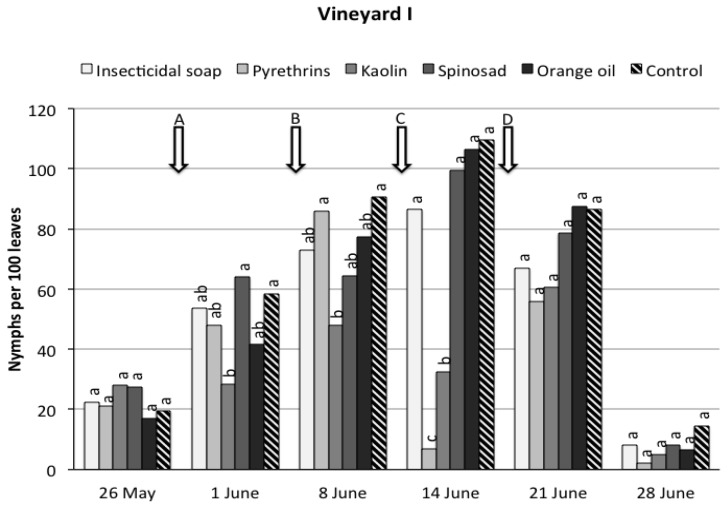
*Scaphoideus titanus* nymphs recorded on grapevine suckers during the sampling period in vineyard I under six different treatments. Within the same date, different small letters above bars indicate significant differences according to *t*-tests on the least square means with Bonferroni correction (α = 0.05). The arrows indicate the application timings of the natural products.

*Vineyard I.* During the sampling period, significant differences were recorded among treatments (*F*_5,19.7_ = 9.52, *p* < 0.0001) (Figure 1). Considering the overall trial period, only kaolin and pyrethrins significantly reduced *S. titanus* nymph densities compared to the control. The time effect was significant (*F*_5,74.2_ = 167.51, *p* < 0.0001) because *S. titanus* numbers were low at the beginning of the trial, increased until the fourth sampling date and were very low on the last sampling date. A significant interaction time*treatment was found (*F*_25,76.5_ = 7.22, *p* < 0.0001) because the efficacy of the treatments varied over time. In the pyrethrin-treated plots, population densities dropped to low numbers after the first application (timing C), but on the subsequent sampling dates they rose to levels similar to the other treatments, despite a second application (timing D). Kaolin-treated plots showed nymph densities lower than the control on the three sampling dates after the first application (timing A), but not on the last sampling date. 

In the spring of 2017, no significant difference in nymph populations was observed between the plots treated with kaolin in the previous year and the control (mean ± SD nymphs per leaf, 1.12 ± 0.41 vs. 1.48 ± 0.18) (*t_6_* = 1.59, *p* = 0.16).

**Figure 2:**
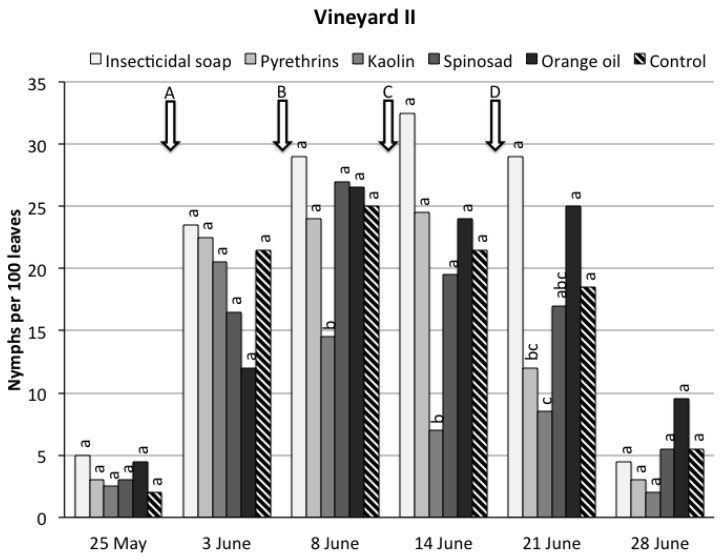
*Scaphoideus titanus* nymphs recorded on grapevine suckers during the sampling period in vineyard II under six different treatments. Within the same date, different small letters above bars indicate significant differences according to *t*-tests on the least square means with Bonferroni correction (α = 0.05). The arrows indicate the application timings of the natural products.

*Vineyard II*. During the sampling period, significant differences were recorded among treatments (*F*_5,36.4_ = 6.13, *p* = 0.0003) (Figure 2). Considering the overall trial period, only kaolin significantly reduced *S. titanus* nymph densities compared to the control. The time effect was significant (*F*_5,83.1_ = 37.9, *p* < 0.0001) because *S. titanus* numbers were low at the beginning of the trial, increased until the fourth sampling date and were very low on the last sampling date. The time * treatment interaction was not significant (*F*_25,84.9_ = 0.85, *p* = 0.67).

In the spring of 2017, no significant difference in nymph populations was observed between the plots treated with kaolin in the previous year and the control (mean ± SD nymphs per leaf, 0.20 ± 0.04 vs. 0.18 ± 0.10) (*t_6_* = 0.72, *p* = 0.50). 

**Figure 3:**
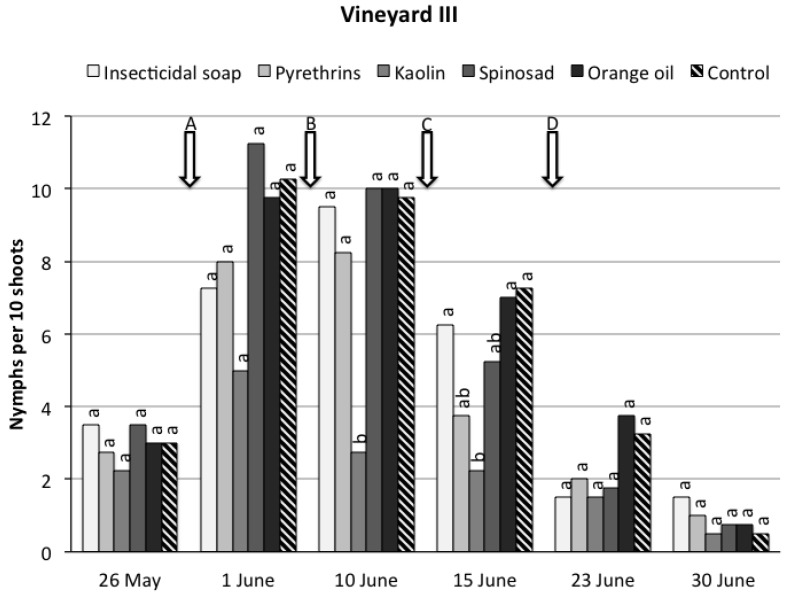
*Scaphoideus titanus* nymphs recorded on grapevine suckers during the sampling period in vineyard III under six different treatments. Within the same date, different small letters above bars indicate significant differences according to *t*-tests on the least square means with Bonferroni correction (α = 0.05). The arrows indicate the application timings of application of the natural products.

*Vineyard III.* During the sampling period significant differences were recorded among treatments (*F*_5,40.1_ = 4.29, *p* = 0.0032) (Figure 3). Considering the overall trial period, only kaolin and pyrethrins significantly reduced *S. titanus* nymph densities compared to the control. The time effect was significant (*F*_5,86.1_ = 34.34, *p* < 0.0001) because *S. titanus* numbers were low at the beginning of the trial, increased until the second sampling date and were very low on the last sampling date. The time * treatment interaction was not significant (*F*_25,87.1_ = 0.91, *p* = 0.59). 

### 3.2. Laboratory Bioassay

**Figure 4:**
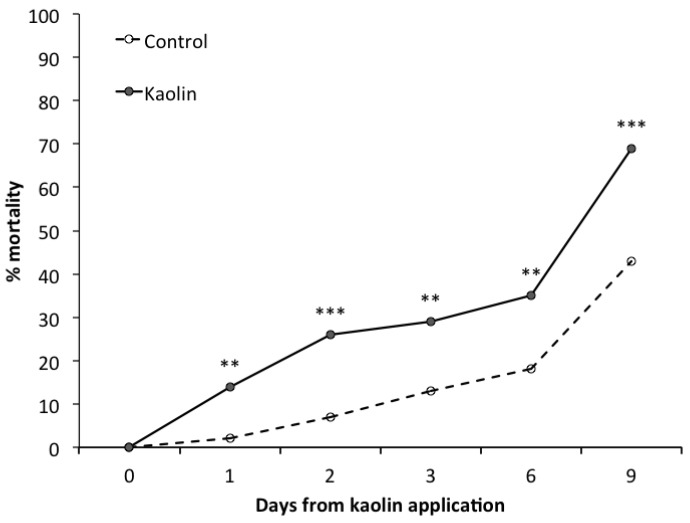
*Scaphoideus titanus* nymph mortality recorded in the laboratory in the kaolin and control. ‘**’ and ‘***’ indicate significant differences for α = 0.01 and α = 0.001, respectively, according to Fisher’s exact test.

One day after the beginning of the bioassay, a significantly higher mortality rate of first-instar and second-instar nymphs of *S. titanus* was observed in the kaolin-treated leaves compared to the control (Figure 4). This difference persisted until the end of the bioassay. The Abbott efficacy calculated for kaolin at the end of this experiment was 46%.

## 4. Discussion and Conclusions

Among the natural products tested in the current study, only kaolin showed an efficacy comparable to that of pyrethrins in the control of *S. titanus* nymphs in vineyards. Based on laboratory data, kaolin increased the mortality of *S. titanus* nymphs. A previous laboratory study showed that kaolin applications increase the mortality of the nymphs of the grapevine leafhopper *E. vitis* [32]. Because kaolin acted against *E. vitis* as a feeding inhibitor, a similar mode of action might be involved with the *S. titanus* nymphs. However, the Abbott mortality of first-instar and second-instar nymphs feeding on kaolin-treated leaves for three days was much lower for *S. titanus* (43.2% in this study) than for *E. vitis* (96.4% in [32]). The lower susceptibility of *S. titanus* can be explained with its larger body size because also for *E. vitis* a lower mortality (48.8% at the third day) occurred when older and then larger nymphs were tested [32]. Moreover, based on nymph samplings carried out in the year following the kaolin applications, the plants coated with this product did not appear to have had any oviposition-deterrent effect.

In all field trials, three kaolin applications against *S. titanus* had an efficacy comparable to two pyrethrin applications (considered as standard procedure in organic Italian vineyards). In these trials, kaolin and pyrethrins reduced nymph numbers with a suboptimal efficacy. Neither of the natural products had significant efficacy in late June when egg hatching was almost complete, and when the aged nymphs disperse along the growing suckers and are able to colonize the upper parts of the grapevine canopy. 

In organic vineyards, a kaolin-based control strategy against *S. titanus* should be preferred to a pyrethrin-based one, particularly when the effects on other pests are considered. Indeed, unlike pyrethrins, kaolin showed a high efficacy against *E. vitis* and *Z. rhamni* [32] and a moderate effect against *L. botrana* both in the laboratory [38] and in the field (Tacoli et al., unpublished data) [39]. Kaolin also has some application advantages over pyrethrins, such as greater persistence and an absence of application-timing issues, due to its use as a preventive control measure from the beginning of *S. titanus* egg hatching. On the other hand, kaolin has been associated with agronomical benefits in vineyards such as reductions in berry-sunburn damage and higher efficiency in water use [40,41]. Nevertheless, side effects of kaolin and pyrethrins on natural enemies should be considered [42,43].

In conventional viticulture, some of the abovementioned benefits of kaolin seem to be less appealing. First of all, the efficacy of kaolin against *S. titanus* is probably much lower than that of synthetic insecticides, as suggested by some studies [23,44]. However, the negative effects of some synthetic insecticides towards natural enemies [45] could counterbalance this gap. 

The moderate effect of three kaolin applications on *S. titanus* suggests that kaolin alone is not enough to achieve the same level of FD control in organic viticulture as obtained with synthetic insecticides in conventional viticulture. Therefore, further research on *S. titanus* control strategies is needed, and in an Integrated Pest Management (IPM) context, alternatives to chemical control should also be considered, such as: (i) conservation and augmentative biological control [6]; (ii) mating disruption based on vibrational disturbance [46,47,48,49]; (iii) symbiotic control based on bacteria that damage the vector or its ability to transmit the phytoplasma causal agent [50,51]; and (iv) push-and-pull strategies [6,52]. Moreover, because *S. titanus* eggs are laid under the bark of two- or more-year-old wood [36], the following cultural practices can be used to reduce *S. titanus* populations: (i) the removal of two-year-old wood from vineyards after winter pruning; and (ii) the removal of suckers growing along the vertical trunk, which are abundantly colonized by nymphs that hatch from the eggs laid into the bark of the trunk [53,54]. In organic vineyards, the integration of these control tools with kaolin applications could increase the efficacy of *S. titanus* management.

## Figures and Tables

**Table 1:** Natural products tested in the vineyards against *Scaphoideus titanus*.

Active Constituent	Commercial Product		Application Rate in Water	Application Timing *
	Name	Formulation		
Kaolin	Surround WP (Tessenderlo Kerley Inc., Phoenix, Arizona, USA)	WP (wettable powder), 95% kaolin	2% *w*/*v*	A, B, C
Orange oil	Prev-Am Plus (Nufarm Italia, Milano, Italy)	SL (soluble liquid), 5.88% orange oil	0.5% *v*/*v*	C, D
Insecticidal soap	Flipper (Dow Agrosciences Italia, Milano, Italy)	SL (soluble liquid), 47.8% potassium salts of fatty acids	>2% *v*/*v*	C, D
Spinosad	Laser (Dow Agrosciences Italia, Milano, Italy)	SC (suspension concentrate), 44.2% pure spinosad	0.02% *v*/*v*	C, D
Pyrethrins	Biopiren Plus (Copyr, Milano, Italy)	EC (emulsifiable concentrate), 2% pure pyrethrins	0.16% *v*/*v*	C, D

(*) A, B, C, D refer to the appearance of first-instar, second-instar, third-instar and fourth-instar nymphs of *S. titanus*, respectively.
